# Extraction and implant restoration of an inverted impacted anterior tooth with severe crown-root dilaceration: a 4-year follow-up case report

**DOI:** 10.1186/s12903-026-07834-5

**Published:** 2026-02-17

**Authors:** Wenting Jiang, Jun Li, Changfu Xie, Mingdong Yan

**Affiliations:** 1 Department of Oral Implantology, School and Hospital of Stomatology, Fujian Key Laboratory of Oral Diseases & Fujian Provincial Engineering Research Center of Oral Biomaterial & Stomatological Key Lab of Fujian College and University, Fujian Medical University, NO.246 Yangqiao Road, Gulou District, Fuzhou, 350002 China; 2 Fujian Key Laboratory of Oral Diseases & Fujian Provincial Engineering Research Center of Oral Biomaterial & Stomatological Key Lab of Fujian College and University, School and Hospital of Stomatology, Fujian Medical University, Fuzhou, 350002 China

**Keywords:** Dilaceration, Alveolar ridge preservation, Tenting technique, Anterior implant restoration, Delayed implantation

## Abstract

**Background:**

Inverted impaction of anterior teeth with crown-root dilaceration is a rare and clinically challenging developmental anomaly. When orthodontic traction or autotransplantation fails to achieve satisfactory outcomes, extraction followed by implant restoration becomes the preferred treatment option. This approach, however, involves multiple challenges, including the extraction of the inverted impacted tooth, bone defect repair, and aesthetic reconstruction of the anterior dentition.

**Case presentation:**

This single-case report details the treatment process of a 17-year-old patient with a severely dilacerated (approximately 90° crown-root angulation) and inverted impacted maxillary central incisor. The treatment involved extraction, alveolar ridge preservation using a tenting technique to address substantial labial bone deficiency, delayed implant placement, and prosthetic rehabilitation, with a 4-year follow-up. Although a complication related to tenting screw exposure occurred, it was successfully managed, yielding satisfactory functional and aesthetic outcomes.

**Conclusions:**

This 4-year follow-up case demonstrates that alveolar ridge preservation utilizing the tenting technique represents a viable strategy for managing severely dilacerated and impacted anterior teeth. The case highlights the value of multidisciplinary planning, staged bone augmentation, and prosthetically guided soft tissue conditioning in achieving long-term functional and aesthetic success.

**Supplementary Information:**

The online version contains supplementary material available at 10.1186/s12903-026-07834-5.

## Background

Dilaceration is a developmental anomaly of tooth morphology characterized by an abrupt curvature of the crown or root, or an abnormal angulation at the cementoenamel junction. It typically arises from trauma, compression, or other disturbances during the development of the permanent tooth germ, frequently resulting in tooth impaction [1, 2]. The reported prevalence of dilacerated incisors ranges from 0.3% to 1.4% in the general population, while impacted maxillary dilacerated incisors occur in approximately 0.06% to 0.2% of cases. Severely dilacerated teeth, defined by a crown-root angulation exceeding 60°, are particularly uncommon and present considerable clinical challenges [3, 4].

Current management strategies for impacted dilacerated teeth include conservative observation, guided eruption, orthodontic traction, autotransplantation/repositioning, and extraction followed by prosthetic rehabilitation [5–7]. Studies indicate that in labially dilacerated incisors, the severity of curvature correlates positively with patient age, suggesting that delayed treatment is associated with more complex presentations and increased difficulty in intervention [8–10]. Consequently, for patients who do not receive early intervention, extraction with subsequent implant-supported restoration often becomes the treatment of choice when orthodontic traction or replantation is no longer feasible. The key challenges of this approach include labial bone wall defects following extraction of the dilacerated tooth, post-extraction alveolar ridge resorption, and the high aesthetic demands inherent to anterior implant restorations. Additionally, patient age and skeletal maturity must be carefully assessed. In adolescents, incomplete craniofacial development and elevated esthetic expectations necessitate a meticulously staged and individualized treatment planning [11].

The case report presents the successful management of a 17-year-old patient with a severely dilacerated (approximately 90° angulation) and inverted-impacted maxillary central incisor. Given the patient’s age and significant labial bone deficiency, immediate implant placement was contraindicated. A delayed protocol was implemented, involving alveolar ridge preservation (ARP) using a tenting technique to maintain graft space stability. Although postoperative exposure of the tenting screw occurred, the complication was successfully managed, leading to a satisfactory outcome.

The following report details the treatment sequence from extraction and ridge preservation to final prosthetic restoration, and discusses the rationale behind each decision in the context of the aforementioned challenges.

## Case presentation

### Clinical findings

In July 2019, a 17-year-old male patient presented to the Department of Implantology at the Affiliated Stomatological Hospital of Fujian Medical University, with the chief complaint of unerupted maxillary anterior teeth for ten years. The patient reported a history of trauma to the primary incisors from a fall during childhood but denied any previous oral surgical treatment or prosthetic history. The patient was otherwise healthy, with no systemic diseases or allergies.

**Fig. 1:**
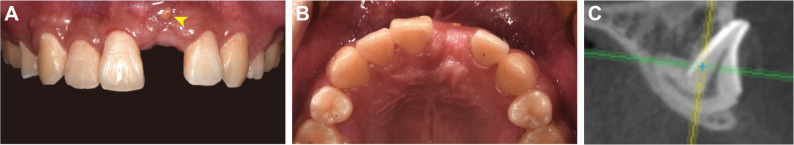
Preoperative intraoral photographs and CBCT images of the maxillary anterior region for tooth 21 extraction. **A** Labial view showing the clinical presentation of the unerupted tooth 21, the yellow arrow indicates partial crown exposure through the thin labial mucosa; (**B**) Occlusal view demonstrating the arch relationship; (**C**) CBCT reconstruction revealing the inverted impaction of tooth 21 with approximately 90° crown-root dilaceration and complete absence of labial bone plate

Clinical examination revealed that tooth 21 was impacted and unerupted, with partial crown exposure through the thin labial mucosa (Fig. 1A-B). Palpation indicated firm, non-tender tissue without adhesion to surrounding structures. The edentulous space was adequate, with no significant tilting of adjacent teeth or overeruption of opposing teeth. Oral hygiene was suboptimal, with localized plaque accumulation and mild gingival inflammation (Fig. 1A). Cone beam computed tomography (CBCT) scanning showed tooth 21 in an inverted impacted position with severe crown-root dilaceration (approximately 90° angulation) and complete absence of the labial bone plate (Fig. 1C).

### Diagnosis

Based on clinical and radiographic findings, the following diagnoses were established: Dilaceration of tooth 21; Inverted impaction of tooth 21; Maxillary dentition defect; Gingivitis.

### Treatment options

Four treatment options were presented for restoring the edentulous space of tooth 21: (1) Orthodontic traction: Not recommended due to the high risk of root exposure, unfavorable crown-to-root ratio, and potential long-term mobility. (2) Extraction with ARP and delayed implant placement: Involved minimally invasive extraction, ARP using a tenting technique, and implant placement after skeletal maturity. (3) Fixed bridge restoration: A conventional prosthesis from teeth 11 to 21, preserving the impacted tooth. (4) Removable partial denture: A conservative prosthetic option for tooth 21. After detailed consultation, the patient chose the second option, which included extraction, ARP, and delayed implant restoration.

### Treatment procedure

#### Control of gingivitis

The patient’s suboptimal oral hygiene was a significant concern. To minimize infection risk and promote healing, professional supragingival scaling and oral hygiene instruction were performed prior to surgery.

#### Tooth extraction and ARP

**Fig. 2:**
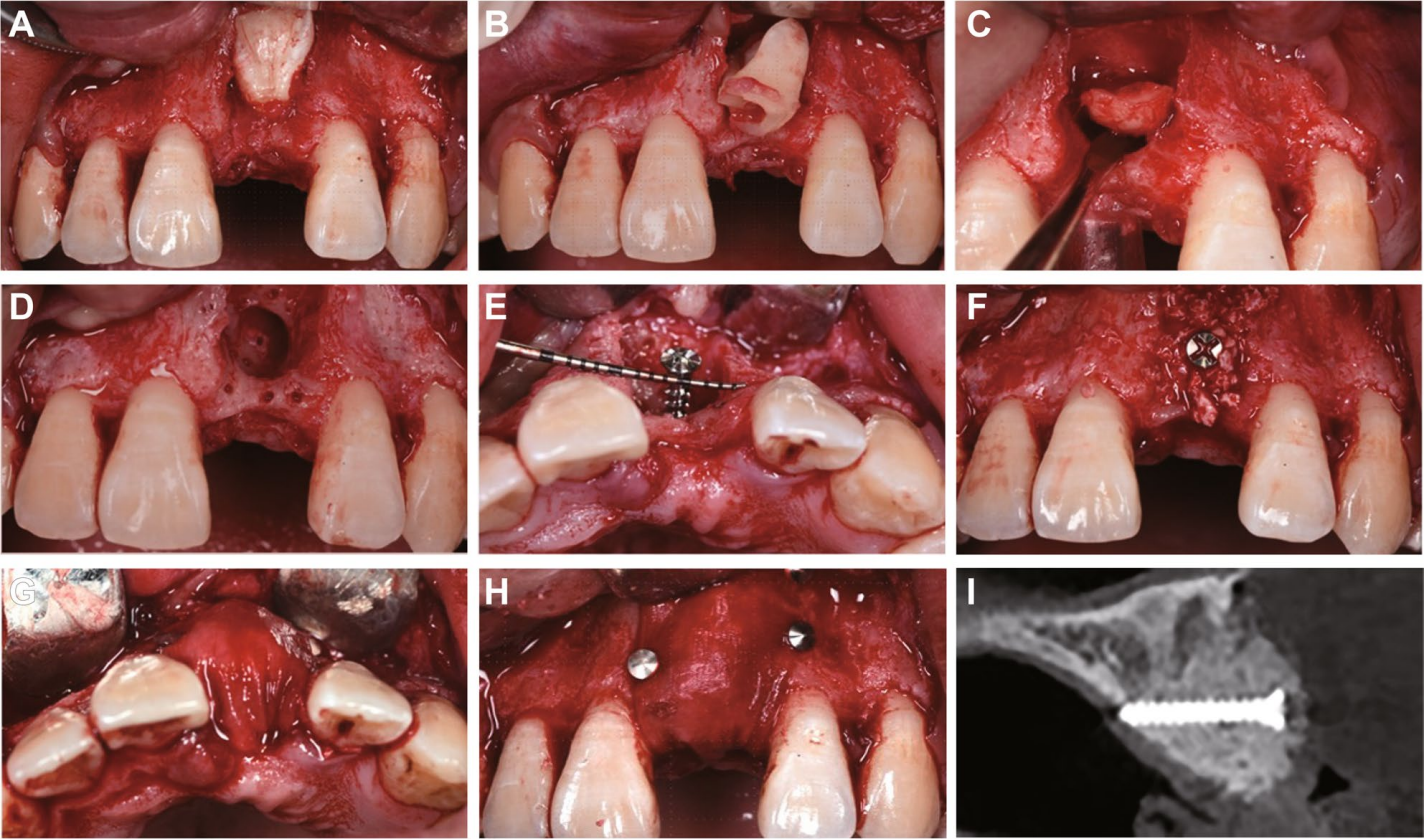
Surgical procedure for tooth 21 extraction and ARP.** A** Full-thickness flap elevation exposing the inverted impacted tooth with missing labial bone; (**B**) Sectioning and removal of the coronal portion; (**C**) Luxation and complete extraction of the root fragment; (**D**) Preparation of nutrient foramina to enhance vascularization; (**E**) Precise placement of a tenting screw (2 mm diameter, 14 mm length) for graft space maintenance; (**F**) Grafting with deproteinized bovine bone matrix (Bio-Oss); (**G**) Application of collagen membrane (Bio-Gide); (**H**) Membrane stabilization with fixation tacks; (**I**) Postoperative CBCT confirming optimal spatial relationships of the tenting screw, maintained graft space, and homogeneous bone substitute distribution

One week after scaling, the extraction of tooth 21 and subsequent ARP procedure were carried out. After obtaining informed consent with detailed explanations of the treatment plan, potential complications and postoperative care, local infiltration anesthesia was administered using articaine. A horizontal incision was made along the alveolar crest of tooth 21, supplemented by oblique incisions distal to teeth 13 and 23. A full-thickness flap was elevated to fully expose the surgical site (Fig. 2A). The tooth was sectioned and carefully removed (Fig. 2B-C). The extraction socket was meticulously debrided to remove residual soft tissue and debris. Multiple nutrient foramina were created in the recipient site to enhance vascularization (Fig. 2D). For the ARP procedure, the tenting screw technique was selected over titanium mesh or autogenous block grafting. Although titanium mesh is effective for managing large defects, it is associated with a higher risk of soft tissue dehiscence and infection, particularly in patients with thin gingival biotype [12, 13]. Autogenous block grafting, although offering excellent osteogenic potential, requires a second surgical site, which was declined by patients and the parent. In contrast, the tenting screw technique, provides stable space maintenance with minimal invasiveness and avoids donor site morbidity, making it particularly suitable for this case with a moderate-sized defect. Based on the buccopalatal distance and the size of the bone defect after extraction of tooth 21, a tenting screw (2 mm in diameter, 14 mm in length) was placed to maintain the graft space (Fig. 2E). The socket was then filled with deproteinized bovine bone mineral (Bio-Oss^®^, Geistlich Pharma) and covered with a single-layer collagen membrane (Bio-Gide^®^, Geistlich Pharma), secured with tacks (Fig. 2F-H). After adequate flap release, tension-free primary closure was achieved. Postoperative CBCT confirmed proper positioning of the tenting screw, adequate graft support, and uniform distribution of bone substitute material beneath the collagen membrane (Fig. 2I). The detailed surgical procedure was illustrated in Video 1 in Supplementary Material.

**Fig. 3:**
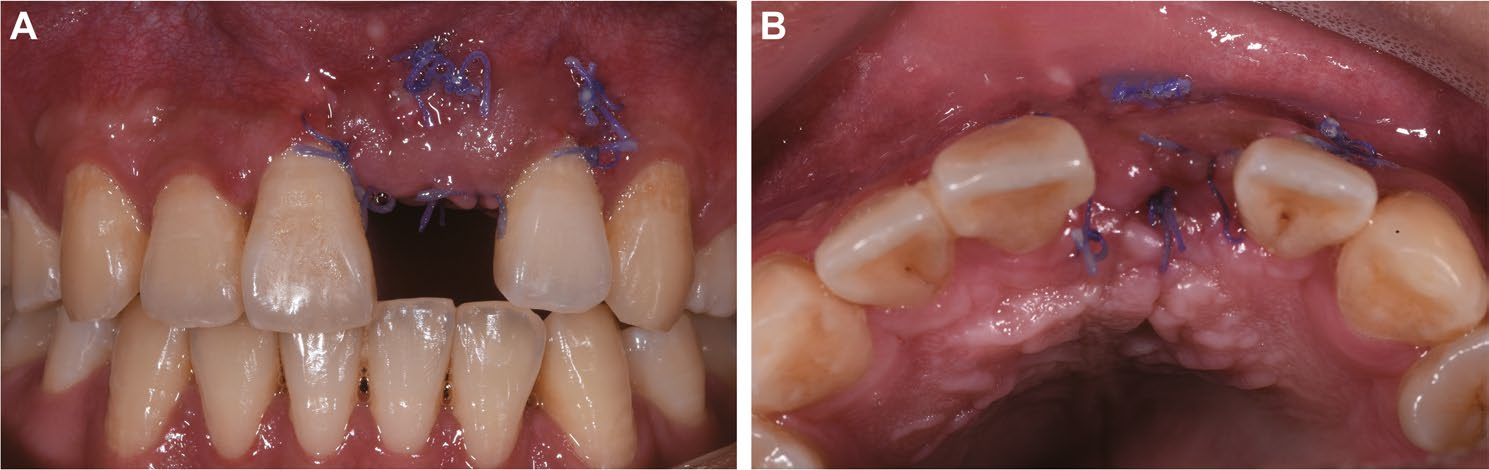
Two-week postoperative healing assessment showing favorable soft tissue recovery. **A** Labial view demonstrating intact gingival architecture; (**B**) Occlusal view confirming proper wound closure

Postoperative instructions included avoiding lip compression, soft diet, cold compression and medications: cefradine (500 mg tid for 3 days), tinidazole (200 mg bid for 3 days), ibuprofen (300 mg as needed for pain), and chlorhexidine mouthwash (tid for 14 days). At the two-week follow-up, the surgical site exhibited satisfactory healing (Fig. 3).

#### Stage I implant surgery

**Fig. 4:**
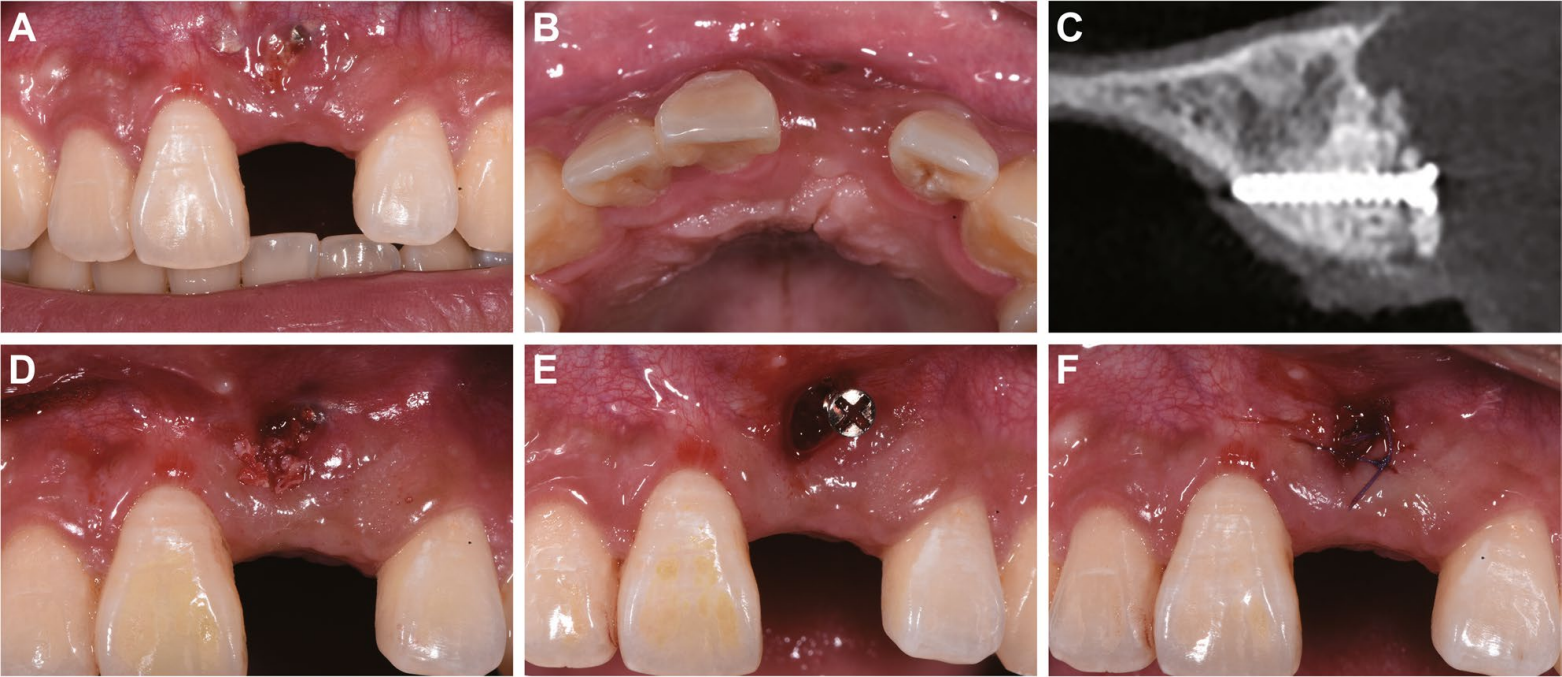
One-year follow-up showing tenting screw exposure and management. **A**-**B** Clinical photographs showing mucosal perforation and exposure of the screw head; (**C**) CBCT imaging displaying successful osteogenesis in most grafted areas with residual particulate material near the screw apex; **D**-**F** Surgical procedure involving debridement of non-integrated particles, screw removal, and primary closure

One year after ARP surgery (July 2020, patient aged 18), screw exposure was noted (Fig. 4A-B). CBCT showed successful osteogenesis in most grafted areas, with minor granular radiopacities around the tenting screw apex (Fig. 4C). Under local anesthesia, the screw was removed, and non-integrated graft particles were debrided before saline irrigation and suturing (Fig. 4D-F).

**Fig. 5:**
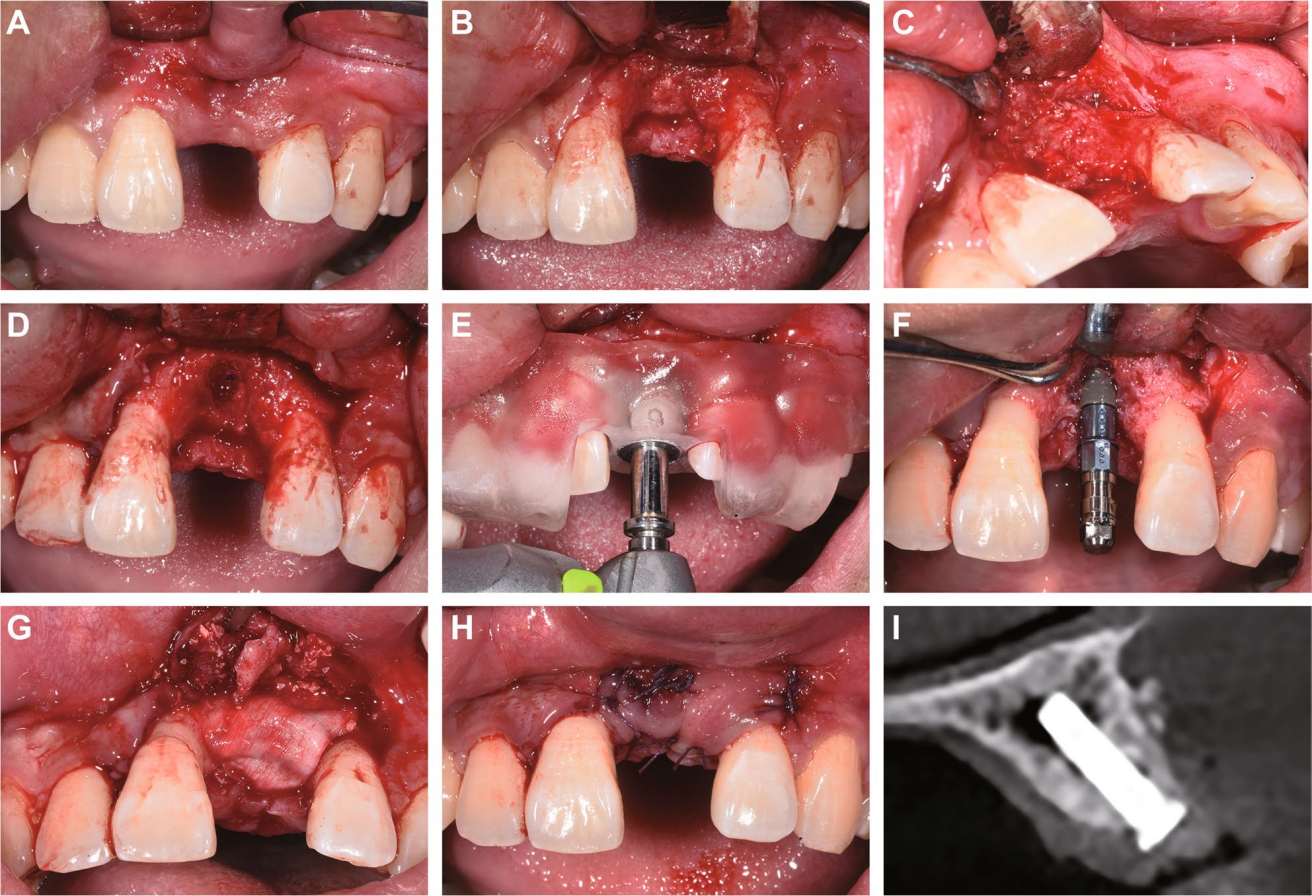
Implant placement with simultaneous GBR procedure. **A** A concave contour was visible on the labial aspect of tooth 21; (**B**) A full-thickness flap was raised; (**C**) Connective tissue was observed at the previous tenting screw exposure site; (**D**) Favorable bone formation was noted after debridement; (**E**) Sequential drilling under an intraoral implant guide; (**F**) Implant placement revealing lack of bone coverage on the labial and cervical aspects; (**G**) Bone grafting and membrane placement; (**H**) Primary wound closure; (**I**) Immediate postoperative CBCT verifying ideal implant positioning and graft distribution

One week later, the implant placement for tooth 21 was performed along with guided bone regeneration (GBR) surgery. The patient was thoroughly briefed on the details of the implant surgery plan and signed an informed consent form. Under local infiltration anesthesia with articaine, horizontal incisions were made along the crest of the alveolar ridge of tooth 21, with oblique incisions made at the mesial axial angle of tooth 11 and the distal axial angle of tooth 22 (Fig. 5A-B). A full-thickness flap was elevated, revealing robust bone formation, although connective tissue was present around the former apex of the tenting screw (Fig. 5C). Following connective tissue removal, the intraoral implant guide was positioned, and drilling was carried out according to the preoperative design (Fig. 5D-E). A bone level implant (diameter 3.3 mm, length 12 mm, Straumann) was placed with an insertion torque of 35 N·cm (Fig. 5F), and a healing abutment was connected. Nutrient foramina were created at the labial bone defect region, and GBR was performed using Bio-Oss and Bio-Gide membrane (Geistlich Pharma), and the wound was tightly sutured (Fig. 5G-H). Immediate postoperative CBCT confirmed that the implant was well-positioned, with uniform filling of bone graft material on the labial and cervical sides of the implant (Fig. 5I).

#### Stage II implant surgery and prosthetic rehabilitation

**Fig. 6:**
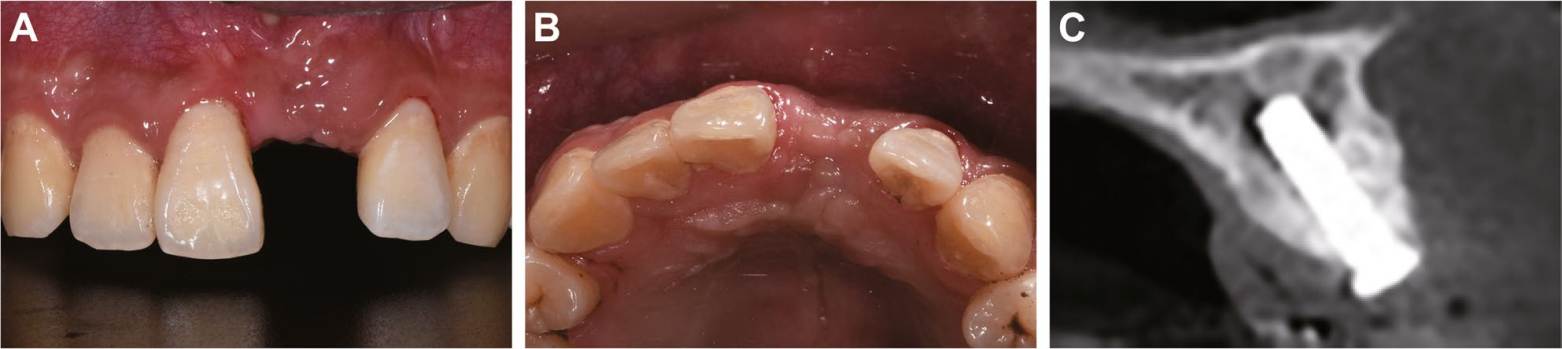
Six-month postoperative evaluation. **A** Healthy peri-implant gingiva without redness or swelling; (**B**) Slight deficiency in the labial contour of tooth 21; (**C**) CBCT demonstrating successful osseointegration and favorable bone formation on the labial grafted area

**Fig. 7:**
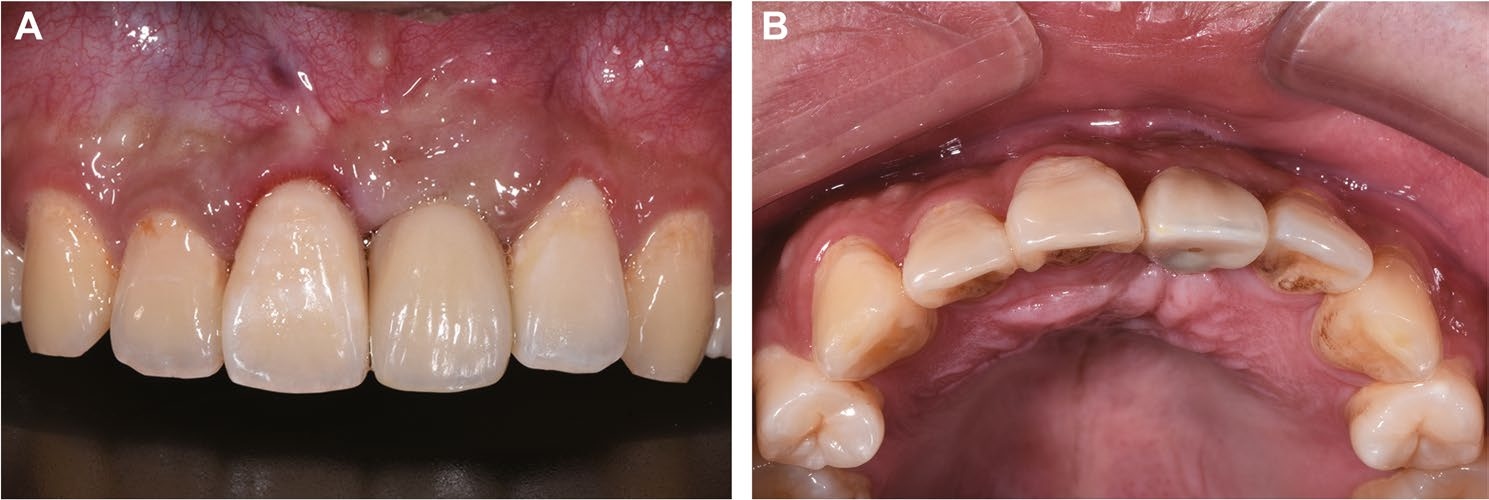
The immediate intraoral photographs after prosthetic restoration of tooth 21.** A **Labial view revealing mismatched gingival margin contours between the implant crown and adjacent teeth; (**B**) Occlusal view showing inadequate labial contour of the implant crown

Six months after the stage I implant surgery in January 2021, clinical and radiographic evaluations confirmed osseointegration and labial bone formation (Fig. 6). Under local anesthesia, the abutment was replaced. Two weeks later, a silicone rubber impression was obtained and the shade selection for the zirconia crown was carefully matched to adjacent dentition. The patient was advised to wear a temporary crown for a period to shape the gingival contour of the implant site for optimal aesthetic outcomes. However, the patient expressed acceptance of potential gingival contour discrepancies between the implant and adjacent teeth and declined the provisional crown for gingival conditioning. The definitive crown was delivered after occlusal adjustment and cemented three weeks later (Fig. 7).

#### Follow-up

**Fig. 8:**
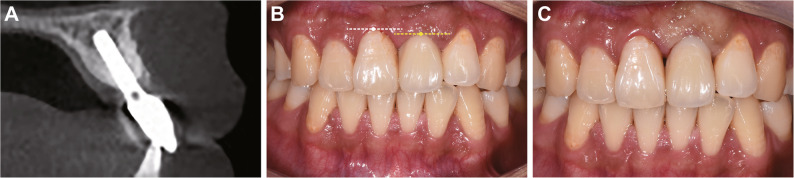
Four-year follow-up assessment. **A** CBCT showing no peri-implant bone resorption, with no significant changes in bone height compared to 4 years ago; (**B**) The gingival contour of the implant crown remaining inconsistent with the contralateral homologous tooth, the gingival zenith of tooth 21 (indicated by the yellow dashed line) is noticeably more apical than that of tooth 11 (marked by the white dashed line); (**C**) Resin bonding was used to modify the prosthesis shape and reshape the gingival contour

**Fig. 9:**
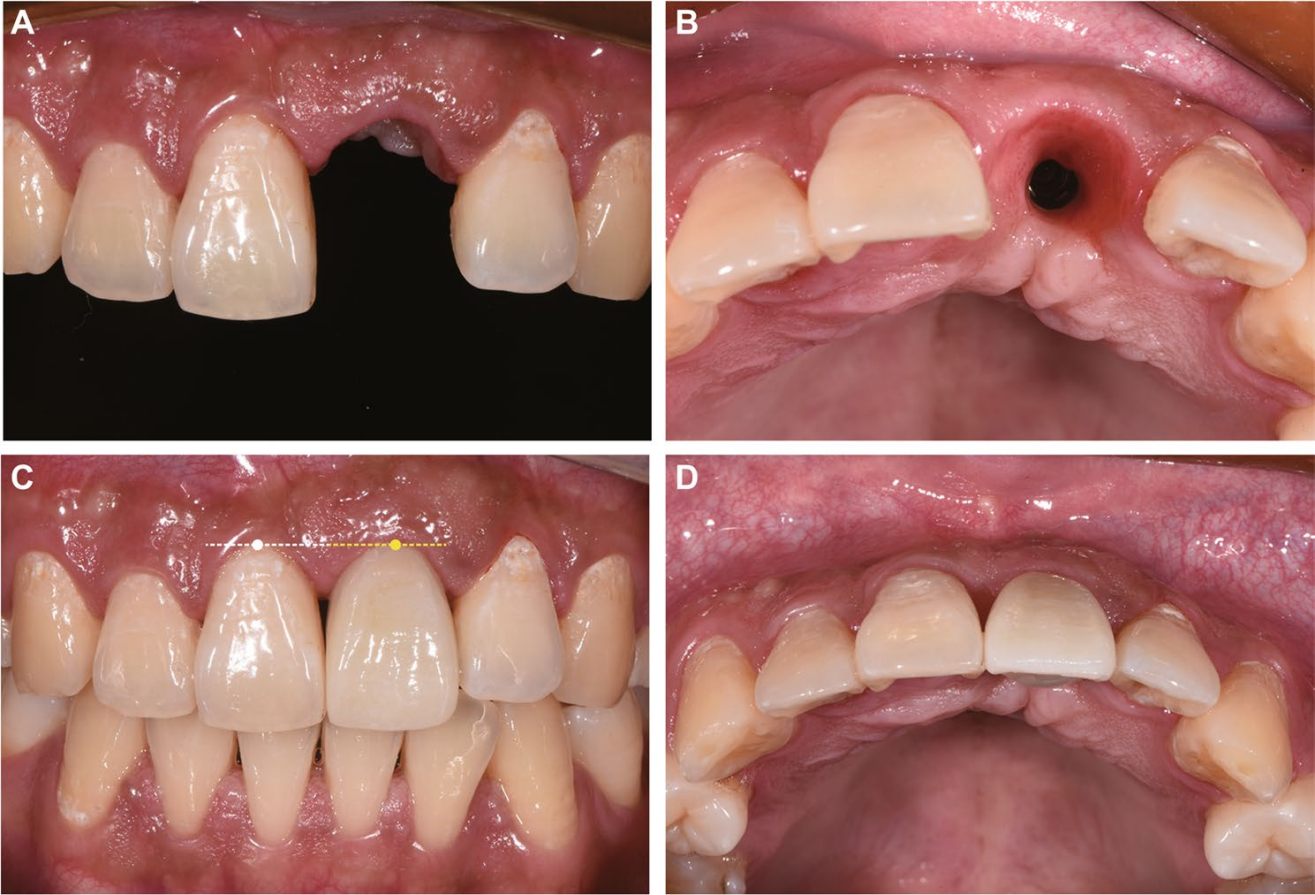
Definitive restoration outcomes following gingival conditioning. **A** Labial view showing harmonious scalloped gingival margins matching adjacent teeth; (**B**) Occlusal view confirming a well-sealed soft tissue interface at the cuff; (**C**) The final crown exhibiting excellent color and morphology matching with adjacent teeth, the gingival zenith of tooth 21 (indicated by the yellow dashed line) aligns closely in height with that of tooth 11(marked by the white dashed line); (**D**) Occlusal view demonstrating full labial contour of the implant crown

At the 4-year follow-up in February 2025, clinical and radiographic examinations demonstrated long-term stability of both hard and soft tissues (Fig. 8A-B). However, discrepancies in gingival margin contour were noted between the restoration and adjacent natural teeth. To address this, the existing zirconia crown was removed and extracorally modified with composite resin to optimize its emergence profile. This adjusted provisional restoration was reinserted to guide soft tissue remodeling (Fig. 8C). Four weeks after gingival conditioning, the implant site demonstrated favorable gingival contour with adequate labial ridge fullness (Fig. 9A-B). A digital impression was obtained and the definitive zirconia crown was delivered, demonstrating perfect integration in terms of color matching, morphological harmony with adjacent teeth (Fig. 9C-D). The patient expressed complete satisfaction with both the functional and aesthetic outcomes of the final restoration.

## Discussion and conclusion

Following tooth extraction, alveolar bone undergoes remodeling in both vertical and horizontal dimensions, with particularly pronounced resorption observed in the maxillary anterior region [14, 15]. Alveolar ridge preservation (ARP) involves the placement of biomaterials into the extraction socket to minimize bone loss and maintain or augment bone volume [16, 17]. It is primarily indicated in sites unsuitable for immediate implant placement or when delayed implantation is planned. As with conventional GBR, successful ARP requires adherence to the “PASS” principles: Primary wound closure, Angiogenesis, Space maintenance, and Stability of the graft [18].

This report presented a case of a severely dilacerated (approximately 90° crown-root angulation) and inverted impacted maxillary central incisor. Following orthodontic assessment, which ruled out conservative treatment, extraction with subsequent implant restoration was selected. Due to the patient’s skeletal immaturity and significant labial bone defect after extraction, immediate implantation was contraindicated. ARP was performed with delayed implant placement planned post-adulthood. The surgical protocol included tooth extraction, meticulous debridement, and intentional perforation of the socket walls to promote bleeding and vascularization. Given the labial defect location—a region highly susceptible to functional stress—graft stability was critical. A tenting screw was therefore placed to maintain space, supported by deproteinized bovine bone mineral and covered with a collagen membrane to ensure containment and tension-free closure. The procedure strictly followed the “PASS” principles. Postoperative CBCT confirmed optimal screw positioning and graft distribution, and healing was uneventful. One-year CBCT revealed substantial new bone formation, providing a viable foundation for implant placement and aesthetic restoration. This case illustrated the value of standardized ARP in complex extraction scenarios.

Tenting screw exposure is a known complication. Although exposure in this case led to localized compromised bone formation around the screw, it did not affect the overall regenerative outcome. The screw was removed promptly, and implant placement proceeded uneventfully one week later. The etiology of screw exposure of the present case may attribute to anatomical and biomechanical factors: the thin labial mucosa, combined with the substantial bone augmentation required, increased localized mucosal tension; frequent lip muscle activity in this region subjected the area to constant friction; the screw head potentially irritated and traumatized the vulnerable mucosa. These cumulative factors likely led to mucosal perforation and exposure. To mitigate such risks, future cases should prioritize enhanced soft tissue coverage, such as the use of a subepithelial connective tissue graft at the time of ARP to reinforce the mucosal barrier. Additionally, screws with wider, flatter heads are recommended to better distribute soft tissue forces. A recent clinical study by Yoon et al. indicated that the use of wide-head screws in horizontal bone augmentation procedures resulted in a screw exposure rate of 1/13 (approximately 7.7%) [19], markedly lower than the previously reported rates of 14.8%–18.2% with conventional standard screws [20–22]. These findings suggest that wide-head screws may offer a comparative advantage in reducing the incidence of screw exposure-related complications. Taken together, in cases of tenting technique-supported bone augmentation, we recommend the following optimized approaches: First, at rigid contact points, soft tissue coverage should be enhanced, such as through palatal connective tissue grafting or similar techniques, to reduce the risk of mucosal perforation; Second, support structures with wider heads should be selected to improve the distribution of soft tissue pressure, thereby decreasing the probability of titanium screw exposure.

For patients with maxillary anterior tooth loss accompanied by labial bone defects, optimal implant aesthetics depend on both alveolar ridge reconstruction and careful soft tissue management. An appropriately designed emergence profile helps establish harmonious gingival architecture, enhances aesthetic outcomes, and promotes a protective soft tissue seal that minimizes food impaction and peri-implantitis risk [23, 24]. In this case, although two-stage bone augmentation achieved stable bone support, the initial absence of a provisional crown likely contributed to the gingival discrepancy observed at the 4-year follow-up. The need to subsequently modify the final crown extracoronally to shape peri-implant tissues underscores the importance of provisional restoration for soft tissue conditioning. As emphasized by Menchini-Fabris et al. and Ahamed et al. [25, 26], early or immediate provisionalization is invaluable for guiding soft tissue architecture and achieving predictable aesthetics. Although immediate implantation was not feasible in the present case due to the patient’s age and the extent of labial bone deficiency, more favorable cases may benefit from such proactive restorative approaches.

In conclusion, this 4-year follow-up case illustrates that the tenting technique, combined with ARP, can be a viable approach for managing severe bone defects after extraction of dilacerated and inverted impacted anterior teeth. Despite the favorable clinical outcome, several limitations should be acknowledged. First, the lack of soft tissue grafting may have predisposed to screw exposure. Second, patient-reported outcomes were collected informally through verbal communication rather than using validated instruments. The use of Visual Analog Scale (VAS) or the Oral Health Impact Profile (OHIP) would strengthen future assessments of patient-centered outcomes. Finally, the findings are based on a single-case report, which limits the generalizability of the results. Larger prospective studies are needed to validate the efficacy of the described protocol.

## Supplementary Information

Supplementary Material 1. Video 1 Surgical procedure for tooth 21 extraction and ARP.

## Data Availability

No datasets were generated or analysed during the current study.

## Abbreviations

ARP: Alveolar ridge preservation
CBCT: Cone beam computed tomographic
GBR: Guided bone regeneration
mg: Milligram
mm: Millimeter
bid: Bis in die
tid: Ter in die

## References

[1] Mockutė G, Klimaitė G, Smailienė D. The morphology of impacted maxillary central incisors: A systematic review. Med (Kaunas). 2022;58(4):462. 10.3390/medicina58040462 PMC9026335 35454301

[2] Jafarzadeh H, Abbott PV. Dilaceration: review of an endodontic challenge. J Endod. 2007;33(9):1025–30. 17931926 10.1016/j.joen.2007.04.013

[3] Uslu O, et al. Prevalence of dental anomalies in various malocclusions. Am J Orthod Dentofac Orthop. 2009;135(3):328–35. 10.1016/j.ajodo.2007.03.030 19268831

[4] Malcić A, et al. Prevalence of root dilaceration in adult dental patients in Croatia. Oral Surg Oral Med Oral Pathol Oral Radiol Endod. 2006;102(1):104–9. 16831681 10.1016/j.tripleo.2005.08.021

[5] Tanaka E, et al. Severe crowding and a dilacerated maxillary central incisor in an adolescent. Angle Orthod. 2006;76(3):510–8. 16637735 10.1043/0003-3219(2006)076[0510:SCAADM]2.0.CO;2

[6] Topouzelis N, et al. Dilaceration of maxillary central incisor: a literature review. Dent Traumatol. 2010;26(5):427–33. 20831640 10.1111/j.1600-9657.2010.00915.x

[7] Pavlidis D, Daratsianos N, Jäger A. Treatment of an impacted dilacerated maxillary central incisor. Am J Orthod Dentofac Orthop. 2011;139(3):378–87. 10.1016/j.ajodo.2009.10.040 21392694

[8] Shi X, et al. The effect of the root dilaceration on the treatment duration and prognosis of unilateral impacted immature maxillary central incisors. Am J Orthod Dentofac Orthop. 2023;163(1):79–86. 10.1016/j.ajodo.2021.08.027 36202699

[9] Sun H, et al. Root morphology and development of labial inversely impacted maxillary central incisors in the mixed dentition: a retrospective cone-beam computed tomography study. Am J Orthod Dentofac Orthop. 2014;146(6):709–16. 10.1016/j.ajodo.2014.07.026 25432251

[10] Bhikoo C, et al. Factors affecting treatment duration of labial inversely impacted maxillary central incisors. Am J Orthod Dentofac Orthop. 2018;153(5):708–15. 10.1016/j.ajodo.2017.09.017 29706219

[11] Elagib MFA, et al. Dental implants in growing patients: A systematic review and meta-analysis. Technol Health Care. 2023;31(3):1051–64. 36502352 10.3233/THC-220581

[12] Cucchi A, et al. Evaluation of complication rates and vertical bone gain after guided bone regeneration with non-resorbable membranes versus titanium meshes and resorbable membranes. A randomized clinical trial. Clin Implant Dent Relat Res. 2017;19(5):821–32. 28745035 10.1111/cid.12520 PMC5655714

[13] Garcia J, et al. Effect of membrane exposure on guided bone regeneration: A systematic review and meta-analysis. Clin Oral Implants Res. 2018;29(3):328–38. 29368353 10.1111/clr.13121

[14] Tan WL, et al. A systematic review of post-extractional alveolar hard and soft tissue dimensional changes in humans. Clin Oral Implants Res. 2012;23(Suppl 5):1–21. 22211303 10.1111/j.1600-0501.2011.02375.x

[15] Farmer M, Darby I. Ridge dimensional changes following single-tooth extraction in the aesthetic zone. Clin Oral Implants Res. 2014;25(2):272–7. 23346895 10.1111/clr.12108

[16] MacBeth ND, Donos N, Mardas N. Alveolar ridge preservation with guided bone regeneration or socket seal technique. A randomised, single-blind controlled clinical trial. Clin Oral Implants Res. 2022;33(7):681–99. 35488477 10.1111/clr.13933 PMC9541021

[17] Mardas N, et al. Is alveolar ridge preservation an overtreatment? Periodontol 2000. 2023;93(1):289–308. 37622682 10.1111/prd.12508

[18] Wang HL, Boyapati L. PASS principles for predictable bone regeneration. Implant Dent. 2006;15(1):8–17. 16569956 10.1097/01.id.0000204762.39826.0f

[19] Yoon NS et al. Retrospective radiographic evaluation of ridge dimensional changes after vertical augmentation using the novel Wide-Head tent pole screw technique. J Funct Biomater. 2025;16(6):215. 10.3390/jfb16060215 PMC12194207 40558901

[20] Yang S, et al. Radiographic evaluation of the tenting screw technique in horizontal alveolar bone augmentation: A retrospective study. Clin Implant Dent Relat Res. 2023;25(3):564–74. 37130799 10.1111/cid.13213

[21] Isik G, et al. Comparison of autogenous block bone graft and screw Tent-Pole techniques for vertical bone augmentation in the posterior mandible: A Split-Mouth randomized controlled study. J Adv Oral Res. 2020;12(1):1–11.

[22] Farias D, et al. Horizontal bone augmentation in the posterior atrophic mandible and dental implant stability using the tenting screw technique. Int J Periodontics Restor Dent. 2021;41(4):e147–55. 10.11607/prd.5137 34328475

[23] Sun TC, Chang TK. Soft tissue management around dental implant in esthetic zone - the current concepts and novel techniques. J Dent Sci. 2024;19(3):1348–58. 39035283 10.1016/j.jds.2024.03.003 PMC11259688

[24] Mueller B, et al. Gingival conditioning with provisional composite veneer prior to final dental restoration: Three-year Follow-up. Oper Dent. 2023;48(3):237–44. 36917629 10.2341/21-116-S

[25] Menchini-Fabris GB, et al. Volume assessment of the external contour around immediate implant with or without immediate tooth-like crown provisionalization: A digital intraoral scans study. J Stomatol Oral Maxillofac Surg. 2023;124(4):101418. 36758898 10.1016/j.jormas.2023.101418

[26] Ahamed SK, et al. Insights into the current management techniques for Peri-Implant gaps: A systematic review. J Clin Med. 2025;14(10):3351. 10.3390/jcm14103351 PMC12111870 40429347

